# Geometric quantum discords of interacting qubits in thermal reservoir

**DOI:** 10.1038/s41598-017-03535-w

**Published:** 2017-06-13

**Authors:** Zhao Li, Xiao-Min Wang, Wu Zhou, Ming-Liang Hu

**Affiliations:** 1 School of Science, Xi’an University of Posts and Telecommunications, Xi’an, 710121 China; 2 Shaanxi Key Laboratory of Information Communication Network and Security, Xi’an University of Posts and Telecommunications, Xi’an, 710121 China

## Abstract

We examined decay dynamics of various geometric quantum discords (GQDs) for two interacting qubits described by the Heisenberg *XY* model and further coupled independently to their respective thermal reservoirs. Compared to the case of noninteracting qubits, our results showed that decay rates of the GQDs can be retarded apparently by properly choosing system parameters of the interaction term. In the long-time limit, the asymptotic values of the GQDs are enhanced evidently by tuning anisotropy of the model and strength of the transverse magnetic field. We further illuminated the relations between different GQDs on characterizing quantum correlations, and observed multiple sudden change behaviors of their dynamics.

## Introduction

Quantum correlations are long-standing concerns of the community of quantum mechanics, and they also play an important role in the emerging field of quantum information science^1^. Historically, quantum correlations in a system have been characterized and quantified from many different aspects, e.g., the widely-studied Bell-type nonlocality^2^ and quantum entanglement^3^. Apart from these progresses, a new framework for characterizing quantum correlations has been established by Henderson and Vedral^4^, as well as by Ollivier and Zurek^5^. They proposed the seminal notion of quantum discord (QD), and subsequently, a series of other discord-like quantum correlation measures were proposed^6–19^. These measures can be categorized roughly into two different families, i.e., the first of those based on the entropy theory^7–10^, and the second of those based on different distance measures of quantum states^11–19^. Other fundamental aspects of QDs such as their interpretation^20–24^ and their local creation property^25–27^ have also been examined in detail.

One of the main reason for researcher's interest in QDs is due to their potential role in quantum information processing tasks such as the deterministic computation with one qubit^28^, remote state preparation^29^, and the quantum advantage of coherent information extraction^30^. In situations like these, QDs were regarded as new physical resources whose roles are different from those of quantum entanglement. Also due to these potential applications and the unavoidable interaction of any quantum system with its surrounding, the control of QDs for various candidates of quantum computation systems have been researcher's concern of recent years^31–43^. The aim of study along this line is to make clear robustness of various QDs^31–35^, and further identify feasible methods for their long-time maintenance^36–41^.

The significance for studying quantum correlation dynamics of open system also lies in that they can help to examine properties of the system such as the critical points of quantum phase transitions^44^, and to understand structures of the corresponding reservoir such as its (non-)Markovianity^45–47^. For noninteracting qubits (e.g., the qubits that are separated far from each other), it was found that in the Markovian region the QDs decay exponentially in time and vanish only asymptotically^36, 48^, while non-Markovianity of the reservoir induces oscillations of the QDs, and their decay can be delayed to some extent by the backflow of information from the reservoir to the system^33, 49, 50^.

When there exists direct interactions between qubits, solution of the evolution equation of the considered system may be more complicated. But just as every coin has two sides, we are also equipped with more adjustable system parameters that can be used to enhance their robustness against decoherence. In particular, the existence of interactions between qubits and their competition with decoherence effects of the reservoir are expected to eliminate to some extent the devastating effects of the reservoir.

In this paper, we investigate such a dissipative model. The relevant system consists of two qubits which are coupled via the Heisenberg *XY* interaction, and every qubit further interacts independently with a thermal reservoir. Based on this setting, we calculated the geometric quantum discords (GQDs) by solving exactly the master equation describing evolution of these two qubits, and compared in detail their dynamical behaviors. The system parameter regions for which decay rates of the GQDs can be slowed down are identified, and singular behaviors of them such as the multiple sudden change (SC) phenomenon and the different orderings of quantum states imposed by different GQDs are observed.

## Results

### Measures of GQD

There are many different discord measures being proposed in the literature^4–17^. We adopt in this work those of the geometric ones. They are generally defined by the minimal distance between *ρ* and the set ${\mathscr{C}}$ of zero-discord states, i.e., $D\,(\rho )={{\rm{\min }}}_{\chi \in {\mathscr{C}}}d(\rho ,\chi )$ ^15, 38^, or by the minimal square distance between *ρ* and the set ${\mathscr{C}}$, i.e., $D\,(\rho )={{\rm{\min }}}_{\chi \in {\mathscr{C}}}{d}^{2}(\rho ,\chi )$ ^11, 13, 17^, where *d* is the distance measure of states. See ref. 12. for a comparison of these different definitions.

To be explicit, the first measure we will adopt is the trace distance discord (TDD) defined as

1 $${D}_{T}(\rho )=\mathop{{\rm{\min }}}\limits_{\chi \in {\mathscr{C}}}\,{d}_{T}(\rho ,\chi ),$$

where ${d}_{T}={\Vert \rho -\chi \Vert }_{1}$ denotes the trace norm^15^, and *D*_*T*_(*ρ*) characterizes the minimal trace distance from the given state *ρ* to the set ${\mathscr{C}}$ of zero-discord states.

The second one is the Hellinger distance discord (HDD) which takes the form^16^

2 $${D}_{H}(\rho )=2\mathop{{\rm{\min }}}\limits_{{{\rm{\Pi }}}^{A}}\,{d}_{H}^{2}(\rho ,{{\rm{\Pi }}}^{A}({\rho }^{1/2})),$$

with ${d}_{H}={\Vert {\rho }^{\mathrm{1/2}}-{{\rm{\Pi }}}^{A}{\rho }^{\mathrm{1/2}}\Vert }_{2}$, ${\Vert \cdot \Vert }_{2}$ is the Hilbert-Schmidt norm, and ${{\rm{\Pi }}}^{A}=\{{{\rm{\Pi }}}_{k}^{A}\}$ is the set of local projective measurements on *A*. Here, *D*_*H*_(*ρ*) equals to twice of the minimal square Hellinger distance from *ρ* to the set ${\mathscr{C}}$ of zero-discord states, and the constant 2 is introduced for normalizing *D*_*H*_(*ρ*) of the two-qubit maximally discordant states. As proved in ref. 16, this definition of HDD can avoid the problem encountered for the GQD measure initially proposed by Dakić *et al*.^11^. Moreover, for any (2 × *n*)-dimensional system, *D*_*H*_ (*ρ*) is also equivalent to the local quantum uncertainty ${\mathscr{U}}\,(\rho )$, i.e., ${D}_{H}(\rho )={\mathscr{U}}\,(\rho )$.

Finally, we will consider the Bures distance discord (BDD) as well. We take the definition of ref. 38. which is given by

3 $${D}_{B}(\rho )=\sqrt{(2+\sqrt{2})/2}\mathop{{\rm{\min }}}\limits_{\chi \in {\mathscr{C}}}\,{d}_{B}(\rho ,\chi ),$$

where ${d}_{B}={[2(1-{F}^{1/2}(\rho ,\chi ))]}^{1/2}$, and $F(\rho ,\chi )={[{\rm{tr}}{({\rho }^{1/2}\chi {\rho }^{1/2})}^{1/2}]}^{2}$ is the Uhlmann fidelity. The constant before min is introduced for the purpose of normalizing *D*_*B*_ *(ρ*) for the maximally discordant two-qubit states. One can check directly that the square of *D*_*B*_ *(ρ*) given in Eq. (3) equals to the normalized ${\tilde{D}}_{B}(\rho )$ given in Eq. (49) of ref. 17. In fact, the two definitions of BDD in refs 17, 38. give qualitatively the same characterization of quantum correlation of a state.

### Solution of the dissipative model

We consider in this paper an exactly solvable model of open quantum system described by the following master equation^51^

4 $$\frac{d\rho }{dt}=-i[\hat{H},\rho ]+\sum _{k=1,2}{ {\mathcal L} }_{k}\rho ,$$

where the first (second) term on the right-hand side of Eq. (4) is the unitary (dissipative) part, and the Hamiltonian

5 $$\hat{H}={J}_{x}{S}_{1}^{x}{S}_{2}^{x}+{J}_{y}{S}_{1}^{y}{S}_{2}^{y}+B({S}_{1}^{z}+{S}_{2}^{z}),$$

with ${S}_{k}^{\alpha }={\sigma }_{k}^{\alpha }/2(\alpha =x,y,z)$, ${\sigma }_{k}^{\alpha }$ is the Pauli operator at site *k*, *B* is the transversal magnetic field, and *J*_*α*_ is the exchange interaction of the two spins. For later use, we denote *J* = (*J*_*x*_ + *J*_*y*_)/2 and Δ = (*J*_*x*_ − *J*_*y*_)/2. Moreover, the dissipative part described by the Lindblad operator is given by

6 $${ {\mathcal L} }_{k}\rho =\sum _{i=\mathrm{1,2}}\frac{{\gamma }_{k}}{2}(2{c}_{i,k}\rho {c}_{i,k}^{\dagger }-{c}_{i,k}^{\dagger }{c}_{i,k}\rho -\rho {c}_{i,k}^{\dagger }{c}_{i,k}),$$

where ${c}_{\mathrm{1,}k}={(\bar{n}+1)}^{\mathrm{1/2}}{\sigma }_{k}^{-}$, ${c}_{\mathrm{2,}k}={\bar{n}}^{\mathrm{1/2}}{\sigma }_{k}^{+}$, and ${\sigma }_{k}^{-}$ $({\sigma }_{k}^{+})$ is the lowering (raising) operator. *c*_1,*k*_ and *c*_2,*k*_ describe respectively, decay and excitation processes of the *k*th qubit due to its interaction with the reservoir, with $\bar{n}$ being the average thermal photons in the reservoir, and *γ*_*k*_ the damping rates which will taken to be equal in the following, i.e., *γ*_1_ = *γ*_2_ = *γ*. Similar models in the study of state transfer^52^ and quantum teleportation^53^ have already been exploited.

For the initial X-type states, Eq. (4) can be solved analytically. For the purpose of presenting the results concisely, we define *ϱ*_*ij*_ = *ρ*_*ij*_(*t* = 0), *ϱ*_*ij*±*kl*_ = *ϱ*_*ij*_ ± *ϱ*_*kl*_, and

7 $$\begin{array}{rcl}u & = & 2\sqrt{{{\rm{\Delta }}}^{2}+{B}^{2}},\bar{\gamma }=\gamma (1+2\bar{n}),\varsigma =1/({u}^{2}+{\bar{\gamma }}^{2}),\\ {s}_{1} & = & \varsigma (\bar{\gamma }\,\sin \,ut-u\,\cos \,ut),{s}_{2}=\varsigma (\bar{\gamma }\,\cos \,ut+u\,\sin \,ut),\\ {v}_{1} & = & {B}^{2}+{{\rm{\Delta }}}^{2}\,\cos \,ut,{v}_{2}={{\rm{\Delta }}}^{2}+{B}^{2}\,\cos \,ut,\end{array}$$

then elements of the evolved *ρ*(*t*) are as follows:

8 $$\begin{array}{ccc}{\rho }_{11}(t) & = & (i{\rm{\Delta }}{a}_{1}+\gamma \bar{n}{a}_{2}+{a}_{3}){e}^{-2\bar{\gamma }t},\\ {\rho }_{44}(t) & = & {\rho }_{11}(t)-{b}_{1}-{\sum }_{m=1}^{3}{c}_{m}{d}_{m}{e}^{-\bar{\gamma }t},\\ {\rho }_{22}(t) & = & \frac{1}{2}[1-{\rho }_{11}(t)-{\rho }_{44}(t)+({\varrho }_{22-33}\,\cos \,2Jt+i{\varrho }_{23-32}\,\sin \,2Jt){e}^{-\bar{\gamma }t}],\\ {\rho }_{14}(t) & = & \frac{1}{2}{\sum }_{m=1}^{3}{c}_{m}{e}_{m}{e}^{-\bar{\gamma }t}+\frac{1}{2}({b}_{2}+{b}_{3}),\\ {\rho }_{23}(t) & = & \frac{1}{2}[{\varrho }_{23+32}+{\varrho }_{23-32}\,\cos \,2Jt+i{\varrho }_{22-33}\,\sin \,2Jt]{e}^{-\bar{\gamma }t},\end{array}$$

and ${\rho }_{41}(t)={\rho }_{14}^{\ast }(t)$, ${\rho }_{32}(t)={\rho }_{23}^{\ast }(t)$, *ρ*_33_(*t*) = 1 − *ρ*_11_(*t*) − *ρ*_22_(*t*) − *ρ*_44_(*t*), and the other elements of *ρ*(*t*) remain zero during the evolution. The parameters *c*_1_ = *ϱ*_11−44_ − *b*_1_, *c*_2,3_ = *ϱ*_14±41_ − *b*_2,3_, while

9 $$\begin{array}{ccc}{a}_{1} & = & \frac{2i\xi {s}_{1}}{u}{e}^{\bar{\gamma }t}+\frac{{b}_{3}}{2\bar{\gamma }}{e}^{2\bar{\gamma }t}+{c}_{3}{s}_{2}{e}^{\bar{\gamma }t},\\ {a}_{2} & = & [\frac{(4\xi {s}_{2}+2i{c}_{3}u{s}_{1}){\rm{\Delta }}}{{u}^{2}}+\frac{4B\zeta }{{u}^{2}\bar{\gamma }}]{e}^{\bar{\gamma }t}+\frac{{b}_{1}+1}{2\bar{\gamma }}{e}^{2\bar{\gamma }t},\\ {a}_{3} & = & {\varrho }_{11}-(2\xi +i\bar{\gamma }{c}_{3}){\rm{\Delta }}\varsigma -\frac{\varsigma {{\rm{\Delta }}}^{2}}{2\bar{n}+1}-[\frac{(4\xi \bar{\gamma }+2i{c}_{3}{u}^{2}){\rm{\Delta }}\varsigma }{{u}^{2}}+\frac{4B\zeta }{{u}^{2}\bar{\gamma }}+\frac{{b}_{1}+1}{2\bar{\gamma }}]\gamma \bar{n},\\ {b}_{1} & = & -\frac{(4{B}^{2}+{\bar{\gamma }}^{2})\varsigma }{2\bar{n}+1},{b}_{2}=-\frac{4B{\rm{\Delta }}\varsigma }{2\bar{n}+1},{b}_{3}=-2i{\rm{\Delta }}\gamma \varsigma ,\\ {d}_{1} & = & \frac{4{v}_{1}}{{u}^{2}},{d}_{2}=\frac{4{\rm{\Delta }}B(1-\,\cos \,ut)}{{u}^{2}},{d}_{3}=\frac{2i{\rm{\Delta }}\,\sin \,ut}{u},\\ {e}_{1} & = & {d}_{2}+{d}_{3},{e}_{2}=\frac{4{v}_{2}}{{u}^{2}}-\frac{2iB\,\sin \,ut}{u},{e}_{3}=\,\cos \,ut-\frac{2iB\,\sin \,ut}{u},\end{array}$$

where *ξ* = Δ*c*_1_ − *Bc*_2_ and *ζ* = Δ*c*_2_ + *Bc*_1_.

### Dynamics of the GQDs

By using Eq. (8), we discuss decay dynamics of the GQDs, and show that they exhibit distinct singular behaviors. In particular, we will show that the system parameters of the interaction term can serve as efficient parameters for tuning quantum correlations between the two qubits.

To be explicit, we consider the initial two-qubit Bell states $|{\psi }^{\pm }\rangle =(|01\rangle \pm |10\rangle )/\sqrt{2}$ and $|{\varphi }^{\pm }\rangle =(|00\rangle \pm |11\rangle )/\sqrt{2}$. In fact, as both $|{\psi }^{+}\rangle$ and $|{\psi }^{-}\rangle$ show completely the same behaviors in all cases we considered, and the same for $|{\varphi }^{+}\rangle$ and $|{\varphi }^{-}\rangle$, we give in the following only plots for $|{\psi }^{+}\rangle$ and $|{\varphi }^{+}\rangle$, respectively.

**Figure 1:**
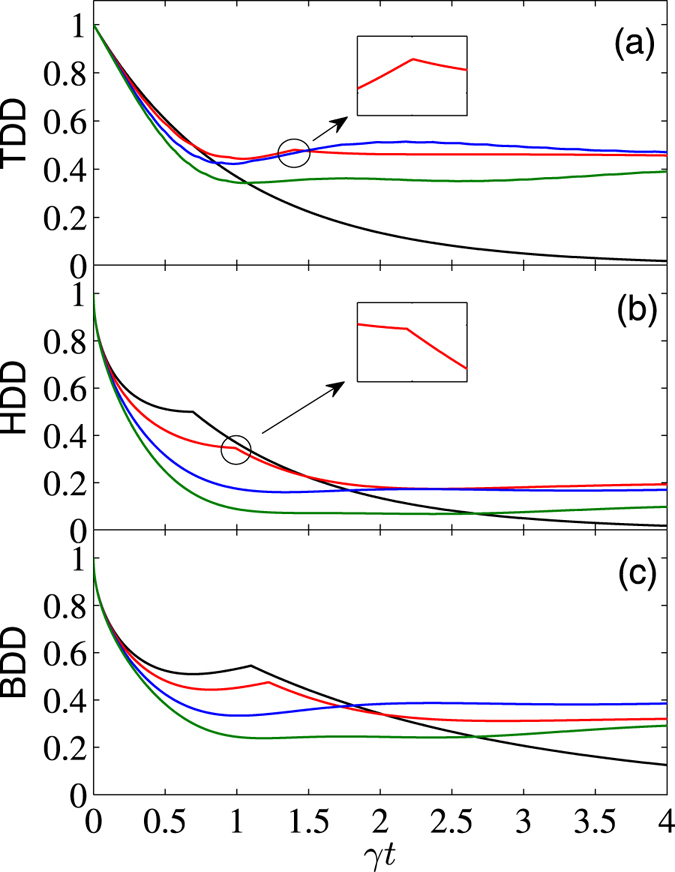
*γt* dependence of TDD, HDD, and BDD (in units of *J*) for the initial state $|{\psi }^{+}\rangle$, with $\bar{n}=0$, *B* = 0, and Δ = 0 (black), 0.3 (red), 0.7 (blue), and 1 (green). Moreover, the black line overlaps with that for noninteracting qubits.

We first investigate the limiting case of zero temperature reservoir, i.e., $\bar{n}=0$. In Fig. 1, we showed the *γt* dependence of *D*_*T*_(*ρ*), *D*_*B*_(*ρ*), and *D*_*H*_(*ρ*) for the initial state $|{\psi }^{+}\rangle$ with *B* = 0 and different anisotropic parameters Δ. When Δ = 0, the TDD can be obtained analytically as *D*_*T*_(*ρ*) = *e* ^−*γt*^. Clearly, it decays smoothly and monotonously with the increasing *γt*. The BDD and HDD show SCs at the critical times $\gamma {t}_{c}\simeq 0.693$ and 1.099, respectively. Mathematically, these SCs are caused by the optimization processes in their respective definitions. That is, the closest states change suddenly when *γt* crosses these critical points, and hence decay rates of the GQDs also changed suddenly. Sometimes, the SC points may also correspond to a transition from the region in which the GQDs are decreased (decreased) to another region in which they turn to be increased (decreased) in time, see, e.g., the inset of Fig. 1(a). Moreover, the BDD also shows a slight increase before the SC point. If one properly enlarges the anisotropy, e.g., Δ = 0.3 denoted by the red curves, all the three GQDs will display SC behaviors, which still occur at different instants ($\gamma {t}_{c}\simeq 1.406$, 0.929, and 1.222, respectively). This fact shows that the three GQD measures give different characterizations of the quantum correlation feature of a system, both qualitatively and quantitatively. In another word, the three GQDs can impose different orderings of quantum states. If one continues enlarging strength of the anisotropic parameter (e.g., Δ = 0.7 and 1), the SC behaviors disappear and the three GQDs turn out to be continuous functions of the scaled time *γt*, and they may be increased slightly after the first period of decrease.

For the case of the initial state $|{\varphi }^{+}\rangle$ with $\bar{n}=0$ and *B* = 0, the TDD can be obtained analytically as

10 $${D}_{T}(\rho )=|\frac{i(\sin 2{\rm{\Delta }}t+2{\rm{\Delta }}\cos 2{\rm{\Delta }}t){e}^{-\gamma t}-2i{\rm{\Delta }}}{4{{\rm{\Delta }}}^{2}+1}+{e}^{-\gamma t}|.$$

**Figure 2:**
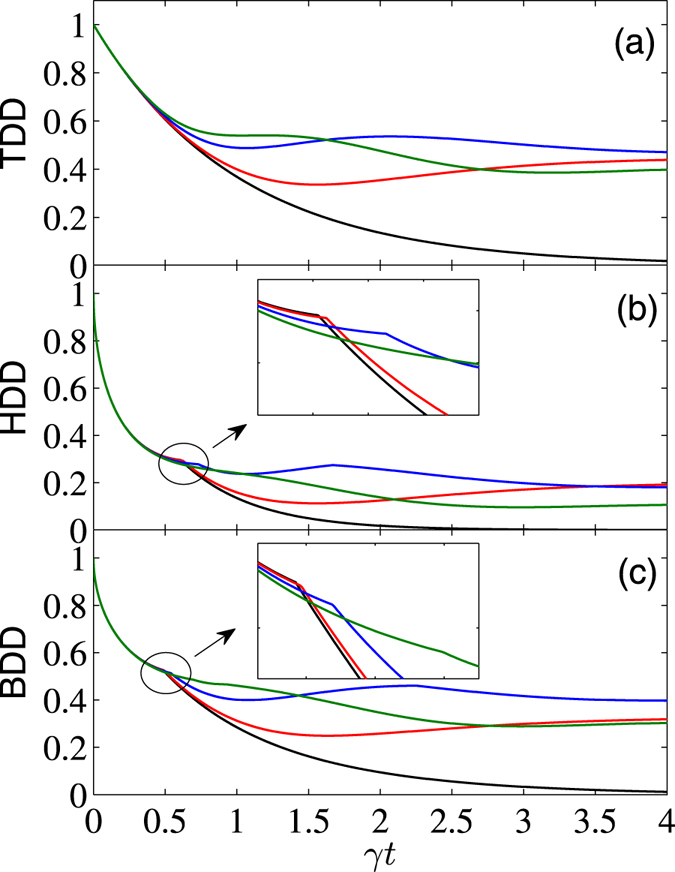
*γt* dependence of TDD, HDD, and BDD (in units of *J*) for the initial state $|{\varphi }^{+}\rangle$, with $\bar{n}=0$, *B* = 0, and Δ = 0 (black), 0.3 (red), 0.7 (blue), and 1 (green). Moreover, the black line overlaps with that for noninteracting qubits.

When Δ = 0, it reduces to *D*_*T*_(*ρ*) = *e* ^−*γt*^, which is completely the same as that for the initial state $|{\psi }^{+}\rangle$. This is understandable as Δ = 0 corresponds to the Heisenberg *XX* model, i.e., *J*_*x*_ = *J*_*y*_, and the Hamiltonian $\hat{H}$ for this special case is invariant by a *π*/2 rotation along the *z* axis. When Δ ≠ 0, due to the existence of sine and cosine terms in Eq. (10), *D*_*T*_(*ρ*) does not behave as a monotonic function of *γt*. But this is not the case for the HDD and BDD. As can be seen from Fig. 2, apart from the case Δ = 1 (in unit of *J*) which corresponds to the Ising model, they undergo SCs during different times of the evolution process. In the long-time limit *t* → ∞, *D*_*T*_(*ρ*) → 2Δ/(4Δ^2^ + 1), which takes the maximum value 0.5 for Δ_*c*_ = 0.5. That is, this is the maximum extent of TDD that can be maintained for $|{\varphi }^{+}\rangle$. Similar phenomenon also happens for the HDD, for which its asymptotic value in the long-time limit is 0.25 (also with Δ_*c*_ = 0.5). For the BDD, however, its asymptotic value of about 0.4186 occurs at a different critical value of Δ, and the numerical simulation shows that it is ${{\rm{\Delta }}}_{c}\simeq 0.61$.

By comparing the curves in Figs 1 and 2, one can also note that for the initial state $|{\psi }^{+}\rangle$, decay rates of the three GQDs are increased by increasing anisotropy of the system in the short-time region, while for the initial state $|{\varphi }^{+}\rangle$, they are nearly the same during the time region *γt* ≲ 0.5. In the long-time limit, the asymptotic values of the GQDs for both the initial states $|{\psi }^{+}\rangle$ and $|{\varphi }^{+}\rangle$ are the same.

**Figure 3:**
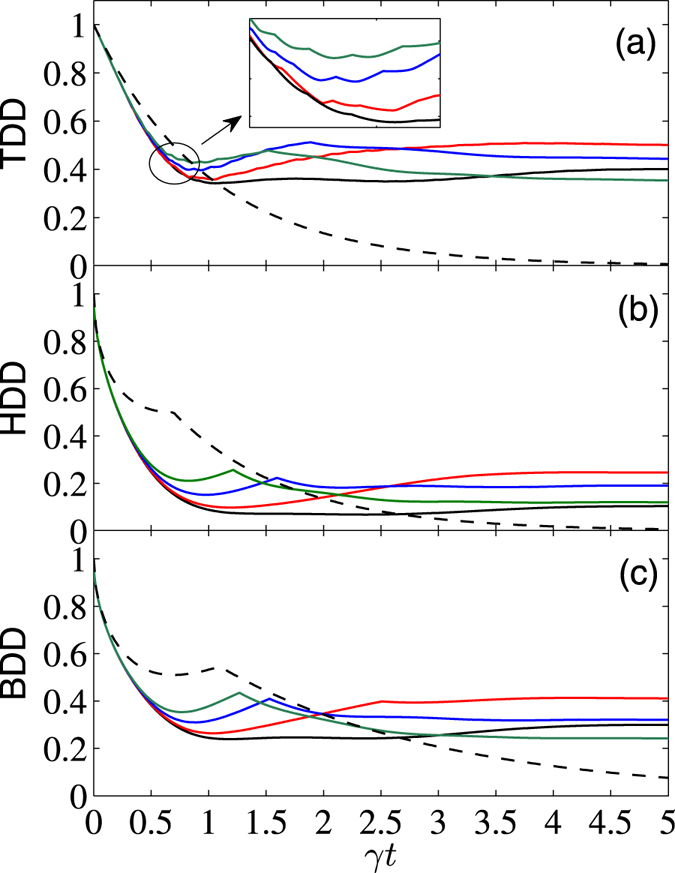
*γt* dependence of TDD, HDD, and BDD (in units of *J*) for the initial state $|{\psi }^{+}\rangle$, with $\bar{n}=0$, Δ = 1, and *B* = 0 (black solid), 0.8 (red solid), 1.6 (blue solid), and 2.4 (green solid). Moreover, the dashed curve shows the case of noninteracting qubits.

**Figure 4:**
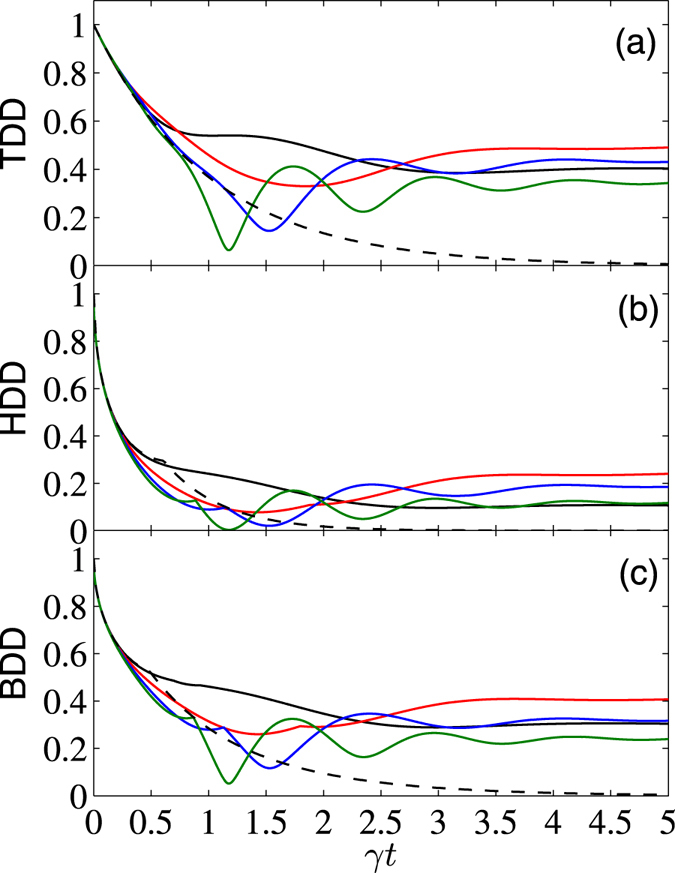
*γt* dependence of TDD, HDD, and BDD (in units of *J*) for the initial state $|{\varphi }^{+}\rangle$, with $\bar{n}=0$, Δ = 1, and *B* = 0 (black solid), 0.8 (red solid), 1.6 (blue solid), and 2.4 (green solid). Moreover, the dashed curve shows the case of noninteracting qubits.

To further see effects of the external magnetic field on control of the GQDs for the considered system, we displayed in Figs 3 and 4 the *γt* dependence of them with fixed Δ = 1 and different values of *B*. From these plots one can see that in the short-time region (*γt* ≲ 0.5 for $|{\psi }^{+}\rangle$, and *γt* ≲ 0.25 for $|{\varphi }^{+}\rangle$), the applied magnetic field does not affect so much the GQDs. But for relatively large *γt*, their decay rates may be slowed down by increasing the strength of *B* for the initial state $|{\psi }^{+}\rangle$, while the opposite cases occur for the initial state $|{\varphi }^{+}\rangle$. We have also calculated numerically the *B* dependence of the asymptotic values of the three GQDs in the long-time limit with different Δ. The results show that when Δ ≥ Δ_*c*_, one can always obtain the same asymptotic values of them as those with Δ = Δ_*c*_ and *B* = 0. The difference is that when Δ increases from Δ = Δ_*c*_, the critical value of *B*_*c*_ also increases linearly. When Δ = 1, we have ${B}_{c}=\sqrt{3}\mathrm{/2}$ for the TDD and HDD, and ${B}_{c}\simeq 0.64$ for the BDD.

Moreover, from the inset of Fig. 3 one can note that the TDD shows multiple SCs during the time evolution process. This is a distinct feature of the TDD dynamics, and the double SCs of the TDD, HDD, and BDD have already been observed in the literature^54, 55^. We have also checked carefully these multiple SCs, and found that they correspond to reciprocating changes of the closest zero-discord states. Note also that the multiple SC phenomenon is different from the decayed oscillations of the GQDs observed in Fig. 4, as the latter are caused by the sine and cosine terms in *ρ*(*t*), and they do not correspond to SCs of the closest zero-discord states. In fact, the first derivatives of the GQDs with respect to *γt* at the SC points are discontinuous, but the GQDs are continuous functions of *γt* at the neighborhood of the extreme points showed in Fig. 4. This constitutes one of the main difference between the SC points and the extreme points of the GQDs.

**Figure 5:**
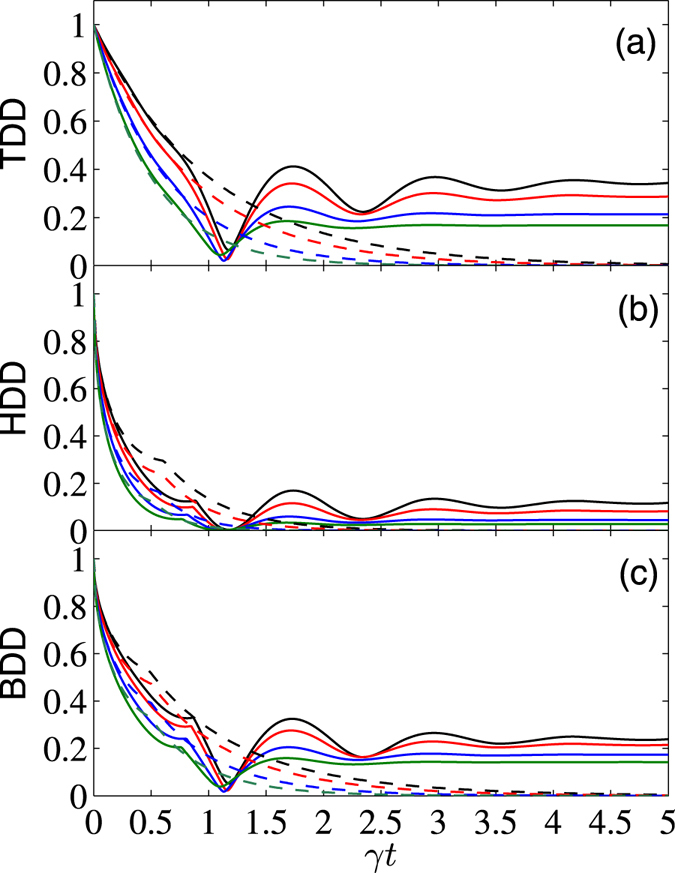
*γt* dependence of TDD, HDD, and BDD (in units of *J*) for the initial state $|{\varphi }^{+}\rangle$, with *B* = 2.4, Δ = 1, and $\bar{n}=0$, 0.1, 0.3, and 0.5 (solid curves from top to bottom). Moreover, the dashed lines show the corresponding plots for noninteracting qubits.

We discussed in the above evolution of the three GQDs in the limiting case $\bar{n}=0$, and observed distinct singular behaviors of them. We now give a short discussion of the finite temperature case. For concise of the paper, we exemplified only plots for the initial state $|{\varphi }^{+}\rangle$ (the case for $|{\psi }^{+}\rangle$ is similar), see Fig. 5. By comparing the curves with different $\bar{n}$, one can note that the decay of the GQDs are accelerated with the increase of the reservoir temperature in nearly the whole time region. This implies that the devastating effects of the thermal reservoir on correlations of the two-qubit system becomes severe and severe with the increase of the reservoir temperature. For the infinite temperature case, decay and excitation occur at the same rate, and the non-diagonal elements of *ρ*(*t*) for any initial state disappear in the long-time limit, thus there are no correlations.

Finally, we present a comparison of the above results with those of the noninteracting two-qubit case (i.e., *J* = Δ = 0). For this case, the reduced density matrix is greatly simplified. For the considered initial Bell states, they are of the Bell-diagonal form, thus analytical solutions of the TDD, HDD, and BDD can be obtained analytically using the results in refs 15, 16, 38. We do not list their explicit expressions here. Alternatively, we showed the corresponding results by dashed lines in Figs 1 to 5 (the dashed lines in Figs 1 and 2 overlap with the black lines). In the short-time region, the combined effects of the dissipative reservoirs and the interaction Hamiltonian induces complex behaviors of the GQDs. As one can see, for the initial state $|{\psi }^{+}\rangle$, the three GQDs for the interacting qubits decay faster than those of the noninteracting ones, while the opposite case occurs for the initial state $|{\varphi }^{+}\rangle$ and weak magnetic field case. In the long-time region, influence of the dissipative term becomes small, while the interaction term of the Hamiltonian turns to dominates, thus decay of the GQDs for the interacting qubits are slower than those for the noninteracting ones for all the considered cases. In particular, in the infinite-time limit, while all the GQDs approach zero for the noninteracting qubits, they maintain finite values for the interacting qubits considered here. This shows potential of the interacting Hamiltonian on enhancing quantum correlations of open system.

## Discussion

We have investigated dynamics of the quantum correlations for an exactly solvable dissipative model, aimed at revealing the interaction Hamiltonian on controlling quantum correlations. The measures of quantum correlations we adopted are the recently introduced TDD, HDD, and BDD, and the central system we considered consists of two qubits which are coupled independently to their respective thermal reservoirs. We compared in detail dynamical behaviors of the three GQDs with different initial states, as well as with different system parameters such as anisotropy of the system, strength of the transverse magnetic field, and the average thermal photons in the reservoir. The results showed that the explicit influence of the reservoir on GQDs are initial-state dependent, and the GQDs can be preserved well compared with those of the noninteracting qubits. When $\bar{n}=0$, the asymptotic values of TDD, HDD, and BDD in the long-time limit are 0.5, 0.25, and of about 0.4186, respectively. These asymptotic values are obtained only in the region of Δ ≥ Δ_*c*_ and when the magnetic field takes certain critical value *B*_*c*_ which increases with the increase of Δ. With the increased temperature, the GQDs will be decreased. Our results also demonstrated the relativity of different GQDs on characterizing quantum correlations. That is to say, they are both quantitatively and qualitatively different. Finally, we observed multiple SCs of the TDD during its evolution process. This complements and extends the observation of the double SCs for its evolution behaviors in open quantum system.

## References

[1] Nielsen, M. A. & Chuang, I. L. *Quantum Computation and Quantum Information* (Cambridge University Press, Cambridge, 2000).

[2] Genovese M (2005). Research on hidden variable theories: a review of recent progresses. Phys. Rep..

[3] Horodecki R, Horodecki P, Horodecki M, Horodecki K (2009). Quantum entanglement. Rev. Mod. Phys..

[4] Henderson L, Vedral V (2001). Classical, quantum and total correlations. J. Phys. A.

[5] Ollivier H, Zurek WH (2001). Quantum discord: a measure of the quantumness of correlations. Phys. Rev. Lett..

[6] Luo S (2008). Quantum discord for two-qubit systems. Phys. Rev. A.

[7] Modi K, Paterek T, Son W, Vedral V, Williamson M (2010). Unified view of quantum and classical correlations. Phys. Rev. Lett..

[8] Luo S (2008). Using measurement-induced disturbance to characterize correlations as classical or quantum. Phys. Rev. A.

[9] Hu ML, Fan H (2012). Dynamics of entropic measurement-induced nonlocality in structured reservoirs. Ann. Phys..

[10] Bai YK, Zhang N, Ye MY, Wang ZD (2013). Exploring multipartite quantum correlations with the square of quantum discord. Phys. Rev. A.

[11] Dakić B, Vedral V, Brukner Č (2010). Necessary and sufficient condition for nonzero quantum discord. Phys. Rev. Lett..

[12] Roga W, Spehner D, Illuminati F (2016). Geometric measures of quantum correlations: characterization, quantification, and comparison by distances and operations. J. Phys. A.

[13] Luo S, Fu S (2010). Geometric measure of quantum discord. Phys. Rev. A.

[14] Luo S, Fu S (2012). Evaluating the geometric measure of quantum discord. Theor. Math. Phys..

[15] Paula FM, de Oliveira TR, Sarandy MS (2013). Geometric quantum discord through the Schatten 1-norm. Phys. Rev. A.

[16] Chang L, Luo S (2013). Remedying the local ancilla problem with geometric discord. Phys. Rev. A.

[17] Spehner D, Orszag M (2013). Geometric quantum discord with Bures distance. New J. Phys..

[18] Luo S, Fu S (2011). Measurement-induced nonlocality. Phys. Rev. Lett..

[19] Hu ML, Fan H (2015). Measurement-induced nonlocality based on the trace norm. New J. Phys..

[20] Madhok V, Datta A (2011). Interpreting quantum discord through quantum state merging. Phys. Rev. A.

[21] Cavalcanti D (2011). Operational interpretations of quantum discord. Phys. Rev. A.

[22] Pati AK, Wilde MM, Usha Devi AR, Rajagopal AK, Sudha (2012). Quantum discord and classical correlation can tighten the uncertainty principle in the presence of quantum memory. Phys. Rev. A.

[23] Hu ML, Fan H (2013). Competition between quantum correlations in the quantum-memory-assisted entropic uncertainty relation. Phys. Rev. A.

[24] Hu ML, Fan H (2013). Upper bound and shareability of quantum discord based on entropic uncertainty relations. Phys. Rev. A.

[25] Hu X, Fan H, Zhou DL, Liu WM (2012). Necessary and sufficient conditions for local creation of quantum correlation. Phys. Rev. A.

[26] Gessner M, Laine EM, Breuer HP, Piilo J (2012). Correlations in quantum states and the local creation of quantum discord. Phys. Rev. A.

[27] Abad T, Karimipour V, Memarzadeh L (2012). Power of quantum channels for creating quantum correlations. Phys. Rev. A.

[28] Datta A, Shaji A, Caves CM (2008). Quantum discord and the power of one qubit. Phys. Rev. Lett..

[29] Dakić B (2012). Quantum discord as resource for remote state preparation. Nat. Phys..

[30] Gu M (2012). Observing the operational significance of discord consumption. Nat. Phys..

[31] Werlang T, Souza S, Fanchini FF, Villas Boas CJ (2009). Robustness of quantum discord to sudden death. Phys. Rev. A.

[32] Hu ML, Fan H (2012). Robustness of quantum correlations against decoherence. Ann. Phys..

[33] Hu ML, Lian HL (2015). Geometric quantum discord and non-Markovianity of structured reservoirs. Ann. Phys..

[34] Aaronson B, Franco RL, Compagno G, Adesso G (2013). Hierarchy and dynamics of trace distance correlations. New J. Phys..

[35] Bai YK, Zhang TT, Wang LT, Wang ZD (2014). Correlation evolution and monogamy of two geometric quantum discords in multipartite systems. Eur. Phys. J. D.

[36] Mazzola L, Piilo J, Maniscalco S (2010). Sudden transition between classical and quantum decoherence. Phys. Rev. Lett..

[37] You B, Cen LX (2012). Necessary and sufficient conditions for the freezing phenomena of quantum discord under phase damping. Phys. Rev. A.

[38] Aaronson B, Franco RL, Adesso G (2013). Comparative investigation of the freezing phenomena for quantum correlations under nondissipative decoherence. Phys. Rev. A.

[39] Xu JS (2010). Experimental investigation of classical and quantum correlations under decoherence. Nat. Commun..

[40] Streltsov A, Kampermann H, Bruß D (2011). Behavior of quantum correlations under local noise. Phys. Rev. Lett..

[41] Hu ML, Tian DP (2014). Preservation of the geometric quantum discord in noisy environments. Ann. Phys..

[42] Hu ML, Fan H (2015). Evolution equation for geometric quantum correlation measures. Phys. Rev. A.

[43] Hu ML, Fan H (2016). Evolution equation for quantum coherence. Sci. Rep..

[44] Hu ML (2010). Disentanglement dynamics of interacting two qubits and two qutrits in an *XY* spin-chain environment with the Dzyaloshinsky-Moriya interaction. Phys. Lett. A.

[45] Shabani A, Lidar DA (2009). Vanishing quantum discord is necessary and sufficient for completely positive maps. Phys. Rev. Lett..

[46] Alipour D, Mani A, Rezakhani AT (2012). Quantum discord and non-Markovianity of quantum dynamics. Phys. Rev. A.

[47] Haikka P, Johnson TH, Maniscalco S (2013). Non-Markovianity of local dephasing channels and time-invariant discord. Phys. Rev. A.

[48] Maziero J, Celeri LC, Serra RM, Vedral V (2009). Classical and quantum correlations under decoherence. Phys. Rev. A.

[49] Fanchini FF, Werlang T, Brasil CA, Arruda LGE, Caldeira AO (2010). Non-Markovian dynamics of quantum discord. Phys. Rev. A.

[50] Wang B, Xu ZY, Chen ZQ, Feng M (2010). Non-Markovian effect on the quantum discord. Phys. Rev. A.

[51] Mintert F, Carvalho ARR, Kuś M, Buchleitner A (2005). Measures and dynamics of entangled states. Phys. Rep..

[52] Hu ML (2010). State transfer in dissipative and dephasing environments. Eur. Phys. J. D.

[53] Hu ML (2011). Teleportation of the one-qubit state in decoherence environments. J. Phys. B.

[54] Montealegre JD, Paula FM, Saguia A, Sarandy MS (2013). One-norm geometric quantum discord under decoherence. Phys. Rev. A.

[55] Hu ML, Sun J (2015). Sudden change of geometric quantum discord in finite temperature reservoirs. Ann. Phys..

